# Instrumental Variable Methods to Target Hypothetical Estimands With Longitudinal Repeated Measures Data: Application to the STEP 1 Trial

**DOI:** 10.1002/sim.70076

**Published:** 2025-04-11

**Authors:** Jack Bowden, Jesper Madsen, Bryan Goldman, Aske Thorn Iversen, Xiaoran Liang, Stijn Vansteelandt

**Affiliations:** ^1^ Novo Nordisk Research Centre (NNRCO) Oxford UK; ^2^ Biostatistics Novo Nordisk Bagsvaerd Denmark; ^3^ Exeter Medical School, University of Exeter Exeter UK; ^4^ Department of Mathematics, Computer Science and Statistics Ghent University Gent Belgium

**Keywords:** causal inference, g‐estimation, instrumental variables, longitudinal data

## Abstract

The STEP 1 randomized trial evaluated the effect of taking semaglutide versus placebo on body weight over a 68‐week duration. As with any study evaluating an intervention delivered over a sustained period, nonadherence was observed. This was addressed in the original trial analysis within the Estimand Framework by viewing nonadherence as an intercurrent event. The primary analysis applied a treatment policy strategy which viewed it as an aspect of the treatment regimen, and thus made no adjustment for its presence. A supplementary analysis used a hypothetical strategy, targeting an estimand that would have been realized had all participants adhered, under the assumption that no post‐baseline variables confounded adherence and change in body weight. In this article, we propose an alternative instrumental variable (IV) method to adjust for nonadherence which does not rely on the same “unconfoundedness” assumption and is less vulnerable to positivity violations (e.g., it can give valid results even under conditions where nonadherence is guaranteed). Unlike many previous IV approaches, it makes full use of the repeatedly measured outcome data, and allows for a time‐varying effect of treatment adherence on a participant's weight. We show that it provides a natural vehicle for defining two distinct hypothetical estimands: the treatment effect if all participants would have adhered to semaglutide, and the treatment effect if all participants would have adhered to both semaglutide and placebo. When applied to the STEP 1 study, they suggest a sustained, slowly decaying weight loss effect of semaglutide treatment.

## 1 Introduction

The growing prevalence of obesity is recognized as one of the most serious health issues of the 21st century. A healthy lifestyle, encompassing a balanced diet and sufficient physical exercise, is a key pillar of the weight management puzzle [1], but many socioeconomic and cultural factors, existing comorbidities and even an individual's genetics [2, 3] make this a much harder prospect for some people than others. Clinical guidelines suggest an increasing role for pharmacological and surgical interventions in the treatment of obesity [4].

Semaglutide was initially developed as a treatment for people living with type II diabetes to improve glycemic control, but its additional effect on body weight led to its subsequent approval for the treatment of obesity. A pivotal trial supporting its use in weight loss was STEP 1 [5]. It randomized 1961 adults with a body mass index of over 30, or 27 with comorbidities, to receive either a weekly 2.4 mg injected dose of semaglutide or a sham placebo injection, as an adjunct to lifestyle intervention. In‐house participant visits occurred at twelve time points, with the primary outcome being mean change in body weight from baseline at week 68.

**FIGURE 1:**
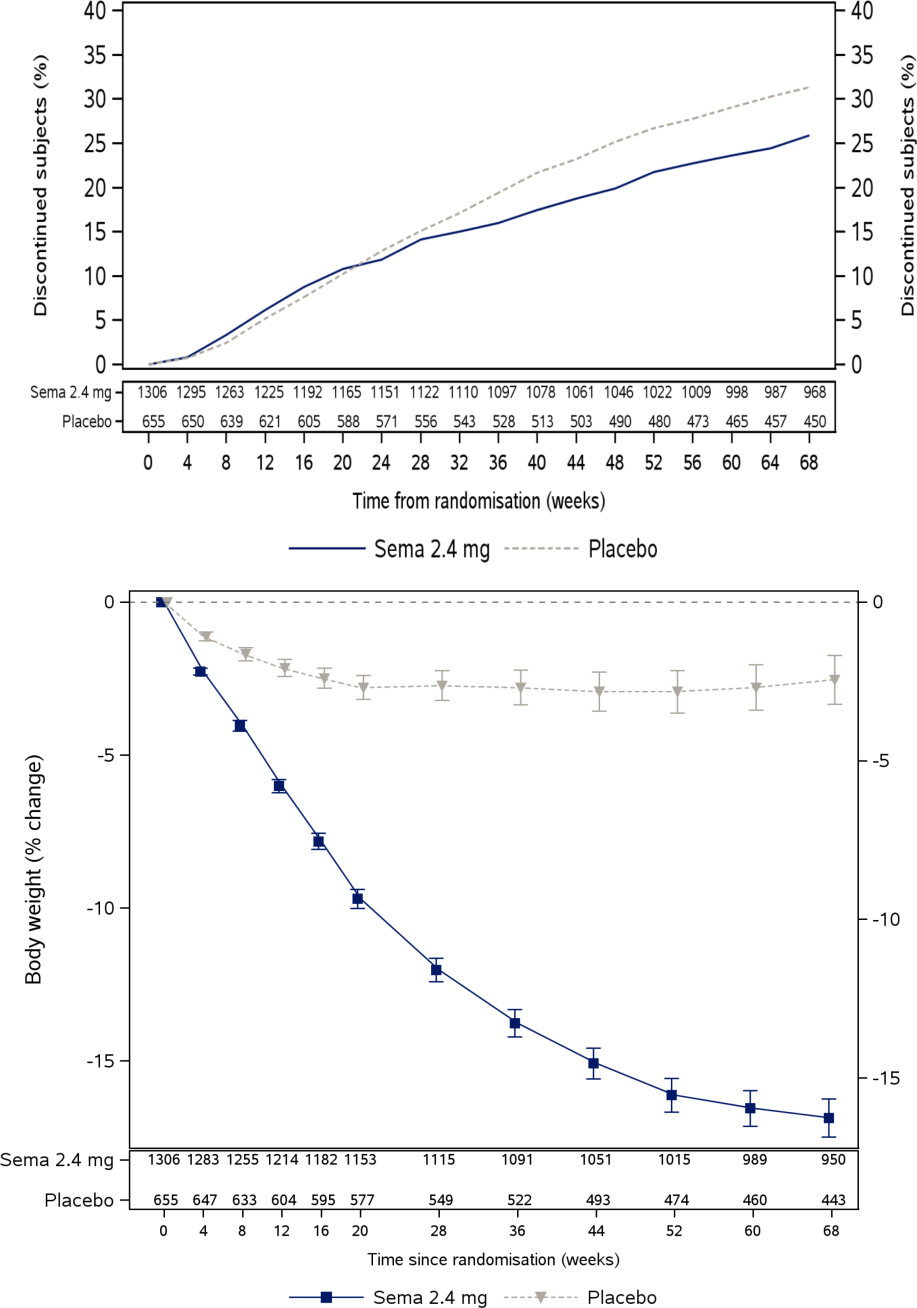
Top: time to discontinuation of the trial product in each arm over 68 week trial duration. Bottom: estimated weight‐loss trajectories in the semaglutide and placebo arms over the 68‐week trial duration of STEP 1 under the MMRM.

As with any trial evaluating an intervention delivered over a sustained period, a degree of nonadherence was observed. By the end of treatment at week 68, approximately 31% of the placebo arm and 26% of the semaglutide arm had experienced a first discontinuation episode (Figure 1 Top), with some pausing treatment momentarily and others stopping treatment permanently. The trial analysis treated this as an “intercurrent event”, which is defined in the International Conference on Harmonisation (ICH) E9 addendum on Estimands [6] as any post‐randomization event that could act to distort the straightforward interpretation of the treatment effect. The addendum provides a set of working strategies for the design, analysis and reporting of clinical trials that accentuates the importance of defining the estimand (the quantity or contrast targeted), who it applies to and how it will be estimated. Specific choices for each of these subcomponents are referred to as an Estimand “strategy”. This has become mandated guidance in industry‐led trials and is referred to in broad terms as the Estimand Framework. The primary *Policy* strategy viewed discontinuation as an integral part of the therapy's total efficacy. The analysis used all weight loss measurements, including those taken after treatment cessation. Approximately 10% of subjects had missing week 68 weight outcomes, which was addressed with multiple imputation, used separately in each treatment arm via a retrieved dropout strategy. It revealed a 14.9% reduction in the treatment arm and 2.4% reduction in the placebo arm, the associated treatment difference being −12.4% (95% CI: −13.4%,−11.5%). A second *Hypothetical* or “trial product” strategy was also used that explicitly adjusted for discontinuation. It used a mixed model for repeated measurements (MMRM) that included participants up until the point of their first discontinuation, and adjusted for baseline body weight. This analysis assumed that missing outcome data was sequentially ignorable given baseline weight, previous adherence status and previous outcome measures [7]. This estimated a 16.9% reduction in the treatment arm and a 2.4% reduction in the placebo arm, the associated treatment difference being −14.4% (95% CI: −15.3%,−13.5%) (Figure 1 bottom).

Instrumental variable (IV) methods have been historically proposed to adjust for nonadherence in clinical trials, where randomization to treatment is the IV. Early examples include the rank‐preserving structural failure time (RPSFTM) model for time‐to‐event data [8, 9] and methods for continuous and binary outcomes based on structural mean models [10, 11, 12] or the identification of compliance classes [13, 14, 15]. The appeal of the IV approach is that it can work even when unmeasured confounding variables simultaneously predict nonadherence and the trial outcome. The problem of confounding is generally associated with analysis of nonrandomized data, for instance, when seeking to estimate the impact of intervening on a disease risk factor in an observational cohort study, or the comparative effectiveness of competing treatments in electronic health record data, which has led to the widespread adoption of IV methods in these fields. Popular approaches include the use of genes as IVs in the first example [16, 17] and physician treatment preference IVs in the second [18, 19].

IV approaches have enjoyed a partially renewed focus in the clinical trials arena since the advent of the Estimand framework in 2017 [6]. Broadly speaking, they can be used to quantify Hypothetical Estimands—the treatment effect in all participants if the intercurrent event had not occurred, or Principal Stratum Estimands—the treatment effect in a subgroup who never experience the intercurrent event under allocation to one or more trial arms [6]. In common with earlier efforts [11, 12, 13], Bowden et al. [20], considered the use of IV methods to target Hypothetical and Principal Stratum Estimands when the intercurrent event is aggregated into a single binary variable and the effect of treatment is considered only for a single outcome measured at the trial's conclusion. This, sometimes defendable but generally incorrect, simplification of the trial data can conveniently side‐step the need to address the issue of time‐varying confounding: that is, where time‐varying measurements on adverse events and participant health influence their future adherence and outcome, which in turn influence adverse events and participant health at later time points. Substantial progress has been made in this regard in the time‐to‐event setting. For example, Ying and Tchetgen Tchetgen [21] and Michiels et al. [22] developed IV‐estimators of a Hypothetical estimand under a structural nested cumulative survival time model, which can incorporate time‐varying adherence and overcome difficulties with the handling of censoring in RPSFTMs [8]. Their estimators are robust to unmeasured time‐varying confounding, as well as more robust to model misspecification than simpler 2‐stage estimators reported in Zuma et al. [23]. Like we, Michael et al. [24] also consider IV‐estimators in a context with time‐varying exposures whose effect on a (continuous) outcome is subject to unmeasured time‐varying confounding. However, their estimators require the availability of a time‐varying IV, therefore not being readily applicable to our motivating study.

Despite this progress, IV approaches are still far less popular than methods that assume the absence of unmeasured confounding. For example, a typical assumption is that participants' decision to discontinue treatment is independent of their counterfactual outcome under continuous treatment or control given a set of baseline and time‐varying covariates [25, 26], or methods that leverage “sequential ignorability” such as the MMRM [7]. Such assumptions, we argue, are often unrealistic as the decision to discontinue treatment likely depends on unmeasured factors that arose during the trial. Furthermore, unlike IV approaches, these methods also rely on the Positivity assumption, which states that all trial participants have a non‐zero probability of experiencing the adherence profiles that form the basis of a given potential outcome contrast (e.g. full adherence to the active treatment and full adherence to placebo). Finally, such approaches can also furnish estimates with p‐values that are smaller than obtained under an intention‐to‐treat (ITT) or Policy estimand analysis. This is regarded in some quarters as undesirable, as it comes at the expense of additional unverifiable assumptions and is potentially open to abuse [22, 27]. IV methods, by contrast, do preserve ITT p‐values by adjusting the effect size to reflect a different causal estimand, but leave its statistical significance unchanged.

In an effort to address this lack of uptake, we propose an IV method to adjust for nonadherence in a trial with longitudinal repeated measures data on adherence status and a continuous trial outcome—in our case weight loss—using the STEP 1 study as an exemplar. As in [22, 23], it allows for a time‐varying causal effect of treatment adherence on a participant's weight and for the presence of unmeasured time‐varying confounding, but unlike [24], our approach uses only the initial randomization indicator as an IV. We show that it provides a natural vehicle for defining two distinct quantities: Hypothetical Estimand 1: the treatment effect that would have been observed had no semaglutide arm participants discontinued and Hypothetical Estimand 2: the treatment effect that would have been observed if no semaglutide *or* placebo arm participants had discontinued. Our proposal leverages the extensive information provided by repeated measures data, incorporating plausible parametric assumptions about the smoothness of the treatment effect over time, to gain insight into the effects of both treatment and placebo. When applied to STEP 1, both Hypothetical Estimand 1 and 2 suggest comparable overall treatment effects for semaglutide in line with original trial analyses, but the individual parameter estimates furnishing Hypothetical Estimand 2 have the cleanest interpretation. This highlights the need to adjust for placebo adherence in our model formulation.

## 2 Methods

For simplicity, we first motivate the technique of G‐estimation [28, 29] by using it to adjust for nonadherence to treatment only, thereby ignoring nonadherence in the placebo arm. This enables the identification and estimation of Hypothetical Estimand 1. An extended version of the model will then be introduced that adjusts for treatment and placebo arm nonadherence, which naturally leads to identification and estimation of Hypothetical Estimand 2.

Let R denote randomization to either the active treatment arm (R=1)—consisting of a semaglutide injection at each study visit, or the control arm (R=0) consisting of a sham placebo injection in place of this. Furthermore, let $Y_{k}$ and $A_{k}$ represent, at time $t_{k}$: an individual's observed percentage weight loss and whether a participant is adherent to the active treatment ($A_{k}=1$) or not ($A_{k}=0$). By design in STEP 1, placebo group participants could not access semaglutide, so according to this particular definition of adherence, $A_{k}=0$ if R=0 for **all** trial time points.

**FIGURE 2:**
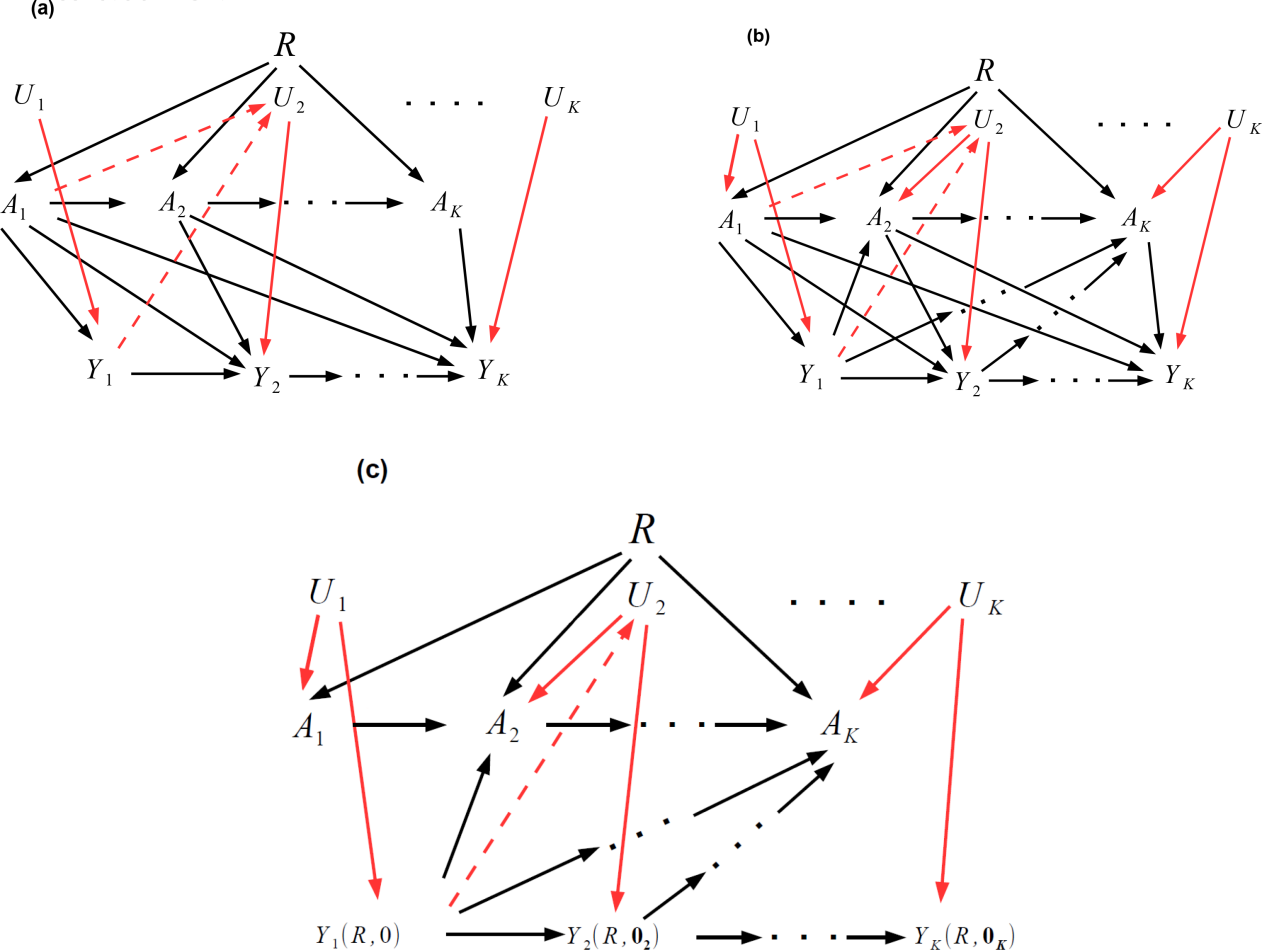
(a) Assumed causal structure for an idealized trial with perfect adherence to treatment (note no confounding of A and Y). (b) Assumed causal structure for the actual trial with only partial adherence. Dashed red lines illustrate the possibility that a participant's earlier treatment adherence and weight may alter later treatment and weight via unmeasured confounders. (c) Approximate visualization of the implied causal structure upon replacing observed treatment arm outcomes with predicted counterfactual outcomes constructed by removing all pathways from A and Y (note this includes direct pathways, as well as pathways via unmeasured confounders). The predicted counterfactual outcomes are then assumed to have the same expectation across randomized groups.

In an idealized trial, all treatment arm individuals would perfectly adhere to their randomly assigned treatment so that, for all time points k, $A_{k}=1$ iff R=1 and $A_{k}=0$ otherwise. This is illustrated in the causal diagram of Figure 2a. Note that in this idealized case, although additional participant‐specific factors, $U_{k}$, (for example side effect history, innate psychological motivation), may influence $Y_{k}$, they **cannot** influence $A_{k}$ as it is uniquely predicted by R in this idealized trial. This guarantees that, at the trial's conclusion, participants who are fully adherent to treatment ($A_{k}$ = 1 for all k) are still exchangeable with participants who were fully nonadherent to treatment ($A_{k}$ = 0 for all k) [7].

In reality, Figure 2a is not an accurate representation of STEP 1 because there *was* treatment arm nonadherence. This means that, at a given time point k, many different participant groups with distinct treatment adherence histories exist, as opposed to the original (intended) two groups of full treatment adherers and no‐treatment adherers. This is problematic because it leaves open the possibility that an individual's:

- Adherence history ${Ā}_{k-1}$=$\left(A_{1},\ldots ,A_{k-1}\right)$
- Weight trajectory ${\bar{Y}}_{k-1}$=$\left(Y_{1},\ldots ,Y_{k-1}\right)$
- Participant factor history ${Ū}_{k}$=$\left(U_{1},\ldots ,U_{k}\right)$

now jointly predict both $A_{k}$ *and* $Y_{k}$. This set up is illustrated in Figure 2b. Taking, for example, data up to time point 2 in the trial and using the rules of d‐separation on Figure 2b [30], to preserve exchangeability between participants with any adherence history ${Ā}_{2}\in \{(0,0),(0,1),(1,0),(1,1)\}$, we would need to adjust for $A_{1}$, $Y_{1}$ and $U_{2}$. That is, all joint predictors of $A_{2}$ and $Y_{2}$ [7]. At any time‐point k, if any components of ${Ū}_{k}$ are either unmeasured or imprecisely measured, it is not possible to satisfy the exchangeability assumption through direct adjustment. We now describe how G‐estimation based on an IV—in our case the initial randomization indicator R—can be used to identify and estimate the treatment effect that would have been seen in STEP 1 if all participants had adhered to the active drug over the full 68 week duration of the trial, had they been randomized to it. Such G‐estimation procedures do not require data on the unmeasured confounder Ū.

### 2.1 Randomization as an IV

For R to be a valid IV for learning the effect of all adherence measures up to time k on the outcome at time k, it needs to satisfy three core assumptions [31, 32]. It must:

- Be a predictor—ideally a strong predictor—of ${Ā}_{k}$ (IV1).
- Not be influenced by any variables that confound the association between $A_{l}$ and $Y_{k}$ for any l≤k (IV2).
- Only influence $Y_{k}$ through ${Ā}_{k}$ (IV3).

Assumption IV1 can be empirically tested in the data. It certainly holds in STEP 1 because, at the 12th and final time point, $Pr({Ā}_{12}=1_{1\times 12}|R=0)=0$ and $Pr({Ā}_{12}=1_{1\times 12}|R=1)\approx 0.74$. Assumption IV2 is automatically satisfied if randomization was appropriately conducted so that a participant's characteristics did not influence their assignment to a given trial arm. This can be partially evaluated by looking at the similarity of aggregated baseline characteristics across trial arms, which were deemed to be satisfied in STEP 1 (see Table 1 in [5]). Assumption IV3 will generally hold if participant care was the same across trial arms, apart from treatment administration, but its validity is heavily dependent on the definition of adherence used in the analysis, as will be discussed subsequently.

Provided R satisfies assumptions IV1–IV3, it can be used to adjust for nonadherence in the following way. Taking ($A_{1},Y_{1}$), the adherence and outcome pair at time $t_{1}$ as an example, Figure 2b shows that their association is confounded by an unobserved variable, $U_{1}$. At time point 1, we can in principle observe the following three potential outcomes $Y_{1}(r,a_{1})$ for each individual:

1. $Y_{1}(1,1)$: the potential outcome at time point 1 if they had been randomized to treatment and adhered.
2. $Y_{1}(1,0)$: the potential outcome at time point 1 if they had been randomized to treatment, but not adhered.
3. $Y_{1}(0,0)$: the potential outcome at time point 1 if they had been randomized to placebo and therefore not adhered to treatment (by design).

In a general context, and following the terminology in [32], IV2 implies that an individual's potential outcomes are independent of the group they were randomized to, or that $Y_{1}(r,a_{1})\;⊥\;R$. Similarly, IV3 implies that $Y_{1}(r,a_{1})$ = $Y_{1}(r^{\prime },a_{1})$ for all distinct pairs $r,r^{\prime }$. In this analysis context $Y_{1}(0,1)$ is not possible since placebo arm patients could not receive semaglutide, so IV3 simplifies to $Y_{1}(1,0)=Y_{1}(0,0)$. In fact, we only require the weaker condition that $Y_{1}(1,0)$ and $Y_{1}(0,0)$ are *mean equivalent*.

Suppose that we were able to predict $Y_{1}(1,0)$ for those for whom we observe $Y_{1}(1,1)$, namely by removing from $Y_{1}$ the unknown effect of $A_{1}$ on $Y_{1}$. This is illustrated in Figure 2c as the removal of all arrows from $A_{1}$ to $Y_{1}$ (in this case a single direct arrow), whose value is denoted as $Y_{1}(R,0)$. This implies that there is now no “open” path from R to $Y_{1}(R,0)$, which in turns means that R and $Y_{1}(R,0)$ are independent, or “d‐separated” [30]. Then since the expected value of $Y_{1}(1,0)$ should be the same as $Y_{1}(0,0)$, the magnitude of this unknown effect can be chosen to force this desired equality under a parametric causal structural model for the effect of $A_{1}$ on $Y_{1}$. This is the principle of G‐estimation. For further reading on the technique's application in clinical trials across a range of settings, see [10, 11, 12, 28, 33].

### 2.2 Adjusting for the Time‐Varying Effect of Nonadherence in the Treatment Arm

More concretely, we now define the causal effect of $A_{1}$ within a structural mean model that links $Y_{1}(1,A_{1})$ to the (possibly) counterfactual predicted outcome $Y_{1}(1,0)$ as:

(1) $$E\left\{Y_{1}|R=1,A_{1}\right\}-E\left\{Y_{1}(1,0)|R=1,A_{1}\right\}=\beta_{1}(1)A_{1}$$

Here, $\beta_{1}(1)$ represents the effect of being adherent to treatment until time point 1 on weight at time point 1 and we say “possibly counterfactual” because $Y_{1}(1,0)$ is directly observed for a proportion of the treatment arm. In a similar fashion, assume that the K−1 potential outcomes for the remaining time points 2,…,K relate to the corresponding observed outcomes $Y_{2},\ldots ,Y_{K}$ via the parametric models:

$$\begin{array}{ll} E\left\{Y_{2}|R=1,{Ā}_{2}\right\}-E\left\{Y_{2}(1,0_{2})|R=1,{Ā}_{2}\right\} & =\beta_{2}(1)A_{1}+\beta_{2}(2)A_{2}\\ & \vdots \\ E\left\{Y_{K}|R=1,{Ā}_{K}\right\}-E\left\{Y_{K}(1,0_{K})|R=1,{Ā}_{K}\right\} & =\overset{K}{\underset{j=1}{\sum }}\beta_{K}(j)A_{j} \end{array}$$

Here, $\beta_{k}(j)$ represents the effect of being adherent to treatment at the jth time point on the outcome at time k and, in a simplification of notation, it is understood that $0_{k}$ denotes a k‐length vector of zeros. Assumptions IV2 and IV3 imply

(2) $$E\left\{Y_{k}(1,0_{k})|R=1\right\}=E\left\{Y_{k}|R=0\right\}$$

for k=1,…,K.

#### 2.2.1 Why is IV1 Needed?

If only outcome data at time point 1 were available, we would estimate $\beta_{1}(1)$ as the value which forced the mean treatment‐free outcomes to be equal across arms, as implied by IV2 and IV3. To facilitate this, note that the estimated coefficient for R in a linear regression of the combined treatment‐free outcomes on R would then be zero. This can only occur when their covariance is zero. The treatment‐free outcome is $Y_{1}-\beta_{1}(1)A_{1}$ for each individual, and therefore:

(3) $$\begin{array}{ll} Cov(R,Y_{1}-\beta_{1}(1)A_{1})=0 & \Rightarrow Cov(R,Y_{1})=\beta_{1}(1)Cov(R,A_{1})\\ & \Rightarrow \beta_{1}(1)=\frac{Cov(R,Y_{1})}{Cov(R,A_{1})} \end{array}$$

From this, we see that assumption IV1 is needed for identification by guaranteeing that the denominator of (3) is non‐zero. The task of jointly estimating the full causal parameter vector $\left(\beta_{1}(1),\ldots ,\beta_{K}(K\right)$) is more complex, and will be explained in detail below, but nevertheless requires assumption IV1 for the same underlying reason.

#### 2.2.2 Hypothetical Estimand 1

An obvious hypothetical estimand can be constructed from the parameters of the structural mean model as the contrast between the expected potential outcome under full treatment adherence up to time K, (${Ā}_{K}=1_{K}$) versus zero treatment adherence, (${Ā}_{K}=0_{K}$):

(4) $$\begin{array}{ll} \text{Hypothetical Estimand 1:} & =E\left(Y_{K}(1,1_{K})-Y_{K}(1,0_{K})\right)\\ \left(\text{By IV3}\;\right) & =E\left(Y_{K}(1,1_{K})-Y_{K}(0,0_{K})\right)\\ & =\overset{K}{\underset{j=1}{\sum }}\beta_{K}(j) \end{array}$$

Equation (4) requires the additional assumption that the effect of full adherence in participants who adhere up to time K is the same as in other participants:

(5) $$\begin{array}{ll} & E\left\{Y_{K}(1,1_{K})-Y_{K}(1,0_{K})|{Ā}_{K}=1_{K},R=1\right\}\\ & \;=E\left\{Y_{K}(1,1_{K})-Y_{K}(1,0_{K})|{Ā}_{K}\neq 1_{K},R=1\right\} \end{array}$$

Assumption (5) is untestable and may well be violated because, for instance, participants who adhere to treatment up to time K may possibly also be more motivated to continue adopting a healthy lifestyle, which might result in a smaller additional effect of treatment. When assumption (5) is violated, then note that (4) can still be interpreted as the effect of full adherence in participants who adhere up to time K, commonly known as the average effect of treatment in the treated (ATT). From Figure 1, we can see that this (observable) subgroup comprises 69% of the treatment arm. Regime history $a=1_{K}$ is the most common in the treatment arm of the STEP 1 study, and provides a clear reason for our chosen definition of Hypothetical Estimand 1 in (4) and our expression of assumption (5). If an alternative regime a was instead of interest, Hypothetical Estimand 1 would need to be modified accordingly and we would then need to replace assumption (5) with

$$\begin{array}{ll} & E\left\{Y_{K}(1,a)-Y_{K}(1,0_{K})|{Ā}_{K}=a,R=1\right\}\\ & \;=E\left\{Y_{K}(1,a)-Y_{K}(1,0_{K})|{Ā}_{K}\neq a,R=1\right\} \end{array}$$

The plausibility of assumption (5) could be increased by conditioning on all predictors of adherence that modified the treatment effect (if they existed) and then appropriately incorporating them into a subsequent causal model, but this is beyond the scope of the present paper. In Section 2.6.2, we discuss the incorporation of baseline covariates into our chosen modeling framework that are predictive of adherence but are not effect modifiers.

### 2.3 Point Estimation for Hypothetical Estimand 1 Parameters

K causal parameters could in theory be estimated under our proposed analysis framework in Section 2.2. This may seem surprising, given that initial randomization is the sole IV. This is because each of the K “adherence‐free” outcomes must be independent of R, each of which enables the identification of a single causal parameter on its own. However, the full causal parameter vector

$$\theta =(\beta_{1}(1),\beta_{2}(1),\beta_{2}(2),\beta_{3}(1),\beta_{3}(2),\beta_{3}(3),\ldots .,\beta_{K}(K))$$

has potentially K(K+1)/2 unique values and therefore cannot all be identified in general. To address this, we propose the following (partially untestable) two‐parameter model that assumes the effect of $A_{k}$ on $Y_{k}$ is constant across all time points k=1,…,K (and equal to β), but its effect thereafter follows the relation:

(6) $$\beta_{k}(j)=\beta \alpha^{t_{k}-t_{j}}$$

Here, $t_{k}$ represents the time that outcome $Y_{k}$ was measured and $t_{k}-t_{j}$ reflects the difference between the earlier treatment time $t_{j}$ and the later treatment time $t_{k}$. If α is less than 1 or greater than 1 this is consistent with the effect of treatment decaying over time or increasing over time, respectively, with the former being more plausible. Estimation of θ proceeds by minimizing a function of the K‐dimensional score equation S(θ) with respect to θ=(β,α) over individuals i=1,…,n, where:

(7) $$\begin{array}{ll} S(\theta ) & =\overset{n}{\underset{i=1}{\sum }}(R_{i}-\bar{R})\sum^{-1}\left(\begin{array}{ccc} Y_{1i} & - & \beta_{1}(1)A_{1i}R_{i}\\ Y_{2i} & - & \left\{\beta_{2}(1)A_{1i}+\beta_{2}(2)A_{2i}\right\}R_{i}\\ & & .\\ & & .\\ & & .\\ Y_{Ki} & - & \overset{K}{\underset{j=1}{\sum }}\beta_{K}(j)A_{ji}R_{i} \end{array}\right) \end{array}$$

Here, $\bar{R}$ is the mean value of R across all participants (e.g. $\approx \frac{1}{2}$ in the case of 1:1 randomization) and ∑ is an arbitrary K×K matrix. For simplicity, we use the identity matrix for ∑ throughout this paper, and opt for a minimization function $S^{T}(\theta )S(\theta )$—that is, the sum of squared contributions of each individual score equation. These choices mean that our analysis strategy can be viewed as a special case of generalized method of moments (GMM) estimation, see for example Equation (1.4) in [34]. The estimation of parameter uncertainty and confidence intervals for Hypothetical Estimand 1 is discussed in Section 2.6.

Although the just‐identified toy example in Section 2.1.1 with 1 time point and 1 parameter is clearly different from the over‐identified system of K estimating equations in (7), its form and motivation are the same: Each individual equation can be viewed as a covariance between randomization and an adherence‐free outcome. However, although in the just‐identified case, we can find the single parameter that sets this covariance precisely to zero, in the over‐identified case, we minimize the sum of the squared covariances across the K equations.

### 2.4 Accounting for Nonadherence in the Treatment and Placebo Arms

A key assumption leveraged by the structural mean model in the previous section, where adherence is defined only with respect to the active treatment, is that counterfactual outcomes $Y_{k}(1,0_{k})$ and $Y_{k}(0,0_{k})$ should be equal in expectation. However, this may very well be violated. For example, subjects who discontinue early under randomization to treatment might perform worse on average than participants who are randomized and adhere to placebo. Furthermore, participants in the placebo arm lost an average of 2.4% of body‐weight in the 68‐week trial. We would not expect sustained weight loss in this population for a 68‐week period outside the confines of the trial, but cannot simply attribute this to a pure placebo effect since 31% of placebo arm participants were nonadherent to the sham injection, with the reasons for this being potentially confounded with their weight loss. Our second G‐estimation strategy therefore attempts to estimate parameters of an extended structural model that equates adherence‐free potential outcomes across trial arms after explicitly subtracting out the effect of adherence to placebo first.

To this end, we now update our definition of adherence so that, at time k,

$$A_{k}=\left\{\begin{array}{ll} 1 & \text{if}\;R=0/1\;\text{and a participant is adherent}\\ & \;\text{to placebo/treatment}\\ 0 & \text{if}\;R=0/1\;\text{and a participant is nonadherent}\\ & \;\text{to placebo/treatment} \end{array}\right.$$

Assumption IV1 holds in STEP 1 because, from Figure 1, it predicts adherence to treatment and placebo across all time points. Assumptions IV2 and IV3 are defined as before, but (we argue below) are more justified with our updated definition of adherence. At time point 1, we can in principle observe the following four potential outcomes $Y_{1}(r,a_{1})$ for each individual:

1. $Y_{1}(1,1)$: the potential outcome for an individual at time point 1 if they had been randomized to treatment and adhered.
2. $Y_{1}(1,0)$: the potential outcome for an individual at time point 1 if they had been randomized to treatment, but not adhered.
3. $Y_{1}(0,1)$: the potential outcome for an individual at time point 1 if they had been randomized to placebo and adhered.
4. $Y_{1}(0,0)$: the potential outcome for an individual at time point 1 if they had been randomized to placebo and not adhered.

As before, IV2 implies that, for each individual, all four potential outcomes should be independent of the trial arm they were randomized to. IV3 implies that $Y_{1}(1,0)$ = $Y_{1}(0,0)$ and is arguably much more plausible. However, whilst both $Y_{1}(1,1)$ and $Y_{1}(0,1)$ are now observable potential outcomes, IV3 does not additionally imply $Y_{1}(1,1)$ = $Y_{1}(0,1)$, since the adherence variable now refers to two different treatments (semaglutide or placebo). Nevertheless, IV2 and IV3 imply K independence conditions that can be exploited for the purposes of estimation. Namely, after a suitable transformation of participants' observed outcomes to adherence‐free outcomes on **both** trial arms:

(8) $$E\left\{Y_{k}(1,0_{k})|R=1\right\}=E\left\{Y_{k}(0,0_{k})|R=0\right\},\;\text{for}\;k=1,\ldots ,K$$

We now define, for the treatment and placebo arm separately, linear structural mean models for the difference between an individual's observed outcome given their treatment and placebo adherence history at time $t_{k}$, and the value it would have taken had these adherence levels been set to 0, as:

(9) $$\begin{array}{ll} E\left\{Y_{k}|R=1,{Ā}_{k}\right\}-E\left\{Y_{k}(1,0_{k})|R=1,{Ā}_{k}\right\} & =\overset{k}{\underset{j=1}{\sum }}\delta_{k}(j)A_{j} \end{array}$$

(10) $$\begin{array}{ll} E\left\{Y_{k}|R=0,{Ā}_{k}\right\}-E\left\{Y_{k}(0,0_{k})|R=0,{Ā}_{k}\right\} & =\overset{k}{\underset{j=1}{\sum }}\gamma_{k}(j)A_{j} \end{array}$$

We parameterize the treatment arm potential outcome with a new term $\delta_{k}(j)$ to reflect the fact that it is now assumed to encapsulate the true treatment effect *and* the additional effect of the placebo at time point j on weight at time point k. Similarly, $\gamma_{j}(k)$ captures only the placebo effect in the full absence of treatment.

#### 2.4.1 Hypothetical Estimand 2

To isolate the pure treatment effect at the end of the trial, we first define the expected difference between observed outcomes under (a) full treatment adherence and no treatment adherence and (b) full placebo adherence and no placebo adherence. The difference between (a) and (b) then forms Hypothetical Estimand 2:

(11) $$\begin{array}{ll} \text{Hypothetical Estimand 2:}\; & =E\left\{Y_{K}(1,1_{K})-Y_{K}(1,0_{K})\right\}\\ & \;-E\left\{Y_{K}(0,1_{K})-Y_{K}(0,0_{K})\right\}\\ (\text{By IV3)} & =E\left\{Y_{K}(1,1_{K})-Y_{K}(0,1_{K})\right\}\\ & =\overset{K}{\underset{j=1}{\sum }}\delta_{K}(j)-\gamma_{K}(j) \end{array}.$$

Furthermore, the last identity relies on assumption (5) as well as

(12) $$\begin{array}{ll} & E\left\{Y_{K}(0,1_{K})-Y_{K}(0,0_{K})|R=0,{Ā}_{K}=1_{K}\right\}\\ & \;=E\left\{Y_{K}(0,1_{K})-Y_{K}(0,0_{K})|R=0,{Ā}_{K}\neq 1_{K}\right\} \end{array}$$

If assumptions (5) and (12) are violated, then we can still interpret Equation (11) as the average effect of full adherence to treatment in those who adhere to treatment up to time K minus the average effect of full adherence to placebo in those who adhere to placebo up to time K. Equation (11) is comparable to the “usual” Hypothetical Estimand in the ICH E9R1 addendum [6], where treatment nonadherence is the only intercurrent event and a hypothetical strategy for this event is considered. In the discussion, we will relate assumptions (5) and (12) to the Principal Stratum approach of Qu et al. (2020) [25] that attempts to estimate the effect in only the subset of participants who would fully adhere under assignment to either treatment or placebo, and the sequential ignorability assumption of Olarte Parra [7].

### 2.5 Point Estimation for Hypothetical Estimand 2 Parameters

For this extended setting, we propose a three‐parameter version of model (6), where:

(13) $$\delta_{k}(j)=\beta \alpha^{t_{k}-t_{j}},\;\gamma_{k}(j)=\left\{\begin{array}{ll} \gamma \; & \text{if}\;j=k,\\ 0\; & \text{otherwise} \end{array}\right.$$

We therefore assume, as before, that the effect of treatment adherence may be long‐lasting, but decays over time, and impose the condition that placebo adherence only has an instantaneous effect at the time it is taken. Although the treatment and placebo arm parameters cannot be simultaneously identified using outcome data at a single time, we leverage the rich availability of repeated outcome measures to estimate the parameters by minimizing $S^{T}(\theta )S(\theta )$ with respect to θ = (β,α,γ), where:

(14) $$\begin{array}{ll} & S(\theta )=\overset{n}{\underset{i=1}{\sum }}(R_{i}-\bar{R})\sum^{-1}\\ & \;\times \;\left(\begin{array}{ccc} Y_{1i} & - & \delta_{1}(1)A_{1i}R_{i}-\gamma A_{1i}(1-R_{i})\\ Y_{2i} & - & \left\{\delta_{2}(1)A_{1i}+\delta_{2}(2)A_{2i}\right\}R_{i}-\gamma A_{2i}(1-R_{i})\\ & & \;.\\ & & \;.\\ & & \;.\\ Y_{Ki} & - & \overset{K}{\underset{j=1}{\sum }}\delta_{K}(j)A_{ji}R_{i}-\gamma A_{Ki}(1-R_{i}) \end{array}\right) \end{array}$$

### 2.6 Further Technical Details on Model Fitting and Inference

#### 2.6.1 Variance Estimation for $\hat{\theta }$

After minimizing a given score equation to obtain $\hat{\theta }$, its variance‐covariance matrix can be approximated by the sandwich expression for a GMM estimator as given in Section 4.3 of [34] 

(15)

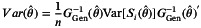


The middle term is estimated by taking the sample variance of the individual score terms at $\theta =\hat{\theta }$, which is a K×K matrix. To calculate the outer term, we execute the following procedure:

1. Calculate the gradient matrix $\left(\frac{\partial S_{i}(\theta )}{\partial \theta }\right)$ for each subject—whose $jl^{th}$ element is the derivative of the $j^{th}$ component of $S_{i}(\theta )$ with respect to the $l^{th}$ element of θ. Note that this is not a square K×K square matrix, but a K×2 or K×3 matrix for our two and three parameter models respectively;
2. Evaluating the result of step 1 at $\hat{\theta }$.
3. Taking the sample average for the result of step 2, so that $G(\hat{\theta })=E\left(\frac{\partial S_{i}(\theta )}{\partial \theta }{|}_{\theta =\hat{\theta }}\right)$.
4. Calculating the generalized inverse 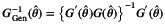.

In the Appendix, we perform a simulation study to verify that the G‐estimation strategies outlined in Section 2.3 and 2.5 can return unbiased estimates for the model parameters furnishing Hypothetical Estimand 1 and 2 when the structural mean model is correctly specified. Moreover, we show that standard errors obtained from sandwich variance formula (15) also accurately quantify the true uncertainty in parameter estimates of the structural mean model. R code is provided for reproducibility.

#### 2.6.2 Incorporating Covariates Into the Model

To improve the efficiency of the G‐estimation approach, we make use of a full set of v baseline covariates C when solving the system of score equations. Taking, for example, Hypothetical Estimand 2, we assume that

$$\begin{array}{ll} E\left\{Y_{k}|R=1,C,{Ā}_{k}\right\}-E\left\{Y_{k}(1,0_{k})|R=1,C,{Ā}_{k}\right\} & =\overset{k}{\underset{j=1}{\sum }}\delta_{k}(j)A_{j},\\ E\left\{Y_{k}|R=0,C,{Ā}_{k}\right\}-E\left\{Y_{k}(0,0_{k})|R=0,C,{Ā}_{k}\right\} & =\overset{k}{\underset{j=1}{\sum }}\gamma_{k}(j)A_{j} \end{array}$$

This approach assumes no effect modification by C (although this can be relaxed). More efficient estimators may then be obtained by using the earlier estimation strategy upon replacing each adherence and weight outcome at time point k by its residual, once the covariate effects have been partialed out. That is we replace:

- $Y_{k}$ with $Y_{k}^{C}=Y_{k}-E(Y_{k}|C)$;
- $A_{k}R_{k}$ with $A_{ik}R_{i}-E(A_{ik}R_{i}|C)$ and $A_{ik}(1-R_{i})\text{ with }A_{ik}(1-R_{i})-E(A_{ik}(1-R_{i})|C)$;

where E(.|C) are obtained as fitted values from a user specified regression model. Misspecification of these regression models does not induce bias, but may result in reduced efficiency (relative to using correct models). We could achieve the same aim by explicitly including covariates within our estimating equations, as opposed to analyzing the covariate‐adjusted residuals. A major advantage of performing this adjustment separately and first, is that uncertainty in the model fitting can then be ignored and the same “master” GMM estimation and variance quantification code can be used, due to the independence of R and C [34] (as long as the models are fitted using ordinary least squares).

#### 2.6.3 Schema for Full Uncertainty Quantification of Weight Loss Trajectories and Hypothetical Estimands in the STEP 1 Data

Fully analytic expressions for the uncertainty in the Hypothetical Estimands are difficult to obtain for two reasons: First, complete weight trajectory data was missing for ≈ 17% of participants in STEP 1. Second, the weight loss trajectories used to calculate the Hypothetical Estimands are nonlinear functions of the estimated model parameters, which is more complicated to approximate via a Taylor series. For example, since participants' weight loss and adherence status was assessed at weeks 2, 4, 8, 12, 16, 20, 28, 36, 44, 52, 60, and 68, Hypothetical Estimand 2 in Equation (11) can be expressed as

$$\begin{array}{ll} & \beta (1+\alpha^{8}+\alpha^{16}+\alpha^{24}+\alpha^{32}+\alpha^{40}+\alpha^{48}+\alpha^{52}\\ & \;+\alpha^{56}+\alpha^{60}+\alpha^{64}+\alpha^{66})-\gamma \end{array}$$

Therefore, following the example of Robins [28], we applied the following uncertainty quantification schema incorporating multiple imputation, analytic sandwich variance approximation and a Monte‐Carlo simulation step.

We used multiple imputation to create a series of complete data sets $D_{q}^{*},q=1\ldots ,m$, each of dimension n×(2K+v+1), where:

$$D_{q}^{*}=(Y_{q1}^{*},\ldots ,Y_{qK}^{*},{Ā}_{K},C,R)$$

Here the superscript 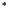 (and lack thereof) denotes variables that vary across imputed data sets. Imputation was performed in four separate subgroups:

- Treatment arm full adherers (R=1 & ${Ā}_{K}=1_{K}$).
- Treatment arm nonfull adherers (R=1 & ${Ā}_{K}\neq 1_{K}$).
- Placebo arm full adherers (R=0 & ${Ā}_{K}=1_{K}$).
- Placebo arm nonfull adherers (R=0 & ${Ā}_{K}\neq 1_{K}$).

We used multiple imputation using chained equations (MICE) software [35] (implemented via predictive mean matching) for this purpose. For each complete data set $D_{q}^{*}$, we calculated ${\hat{\theta }}_{q}^{*}$ by solving the relevant score equation and from that its sandwich variance estimate $\sum_{q}^{*}$. We then used Rubin's rules [36] to calculate pooled point estimates $\hat{\theta }$ and variances $Var(\hat{\theta })$, and the entire covariance matrix $\hat{\sum }$ across the m imputed data sets.

To calculate point estimates and confidence intervals for Hypothetical Estimand 1, we sampled *N*=10,000 parameter values ${\hat{\theta }}_{1},\ldots ,{\hat{\theta }}_{N}$ from a MVN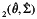 distribution. For each N parameter draws, we constructed the full weight loss trajectory implied by parametric model (6) at time points *k*=1, …,K. From this, we calculated their time‐point specific sample mean and variance. The same procedure was applied for Hypothetical Estimand 2, using the relevant MVN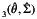 distribution to draw weight loss trajectories from model (13).

## 3 Application to the STEP 1 Trial Data

We now apply our proposed structural mean models to adjust for nonadherence in the treatment arm only (Hypothetical Estimand 1), or in both arms (Hypothetical Estimand 2), using data from the STEP 1 trial. Of the 1961 individuals within STEP 1, 1629 had complete information on their weight outcome history at all 12 time points available, so that $t_{1}=2$, $t_{2}=4$, and so on. For model fitting, multiple imputation using chained equations (MICE) [35] was firstly used to create m=5 data sets via predictive mean matching, making use of a covariate vector C consisting of baseline weight, sex, age, HbA1c and country of study. Out of the 16 countries contributing participants to STEP 1, the largest contingent (38%) were from the United States and the second largest (11%) were from the United Kingdom. Weight outcome data was modeled on the percent change scale, in line with previous publications. To assess the benefit of covariate adjustment in the outcome model and adherence variables (see Section 2.6.2), structural mean models were implemented after adjustment for the full covariate set C and using baseline weight, $Y_{0}$, alone. In each case, this was implemented using simple linear regression models for E(.|C).

When minimizing the relevant score equation S(θ) to furnish the parameters of Hypothetical Estimand 1 and 2, we used the identity matrix in place of ∑. Estimates were calculated separately for each fully imputed data set and then combined using Rubin's rules, as described in Section 2.6.3 [5]. Full results for all analyses are shown in Table 1. Parameter and Hypothetical Estimand estimates are shown to two decimal places, except those for the decay parameter α which are specified to four decimal places—its precise value above or below one warrants additional accuracy due to its subsequent impact on the time‐varying causal effect predictions.

**TABLE 1:** Point estimates, standard errors, 95% confidence intervals and p‐values for the structural mean model parameters (β,α,γ) and the implied Hypothetical Estimands. Results are shown on the percentage reduction scale.

Parameter/Estimand	Estimate	S.E	Lower C.I	Upper C.I	p
Adherence adjustment in treatment arm only: θ=(β,α) (C=$Y_{0}$)					
β	−1.17	0.04	−1.24	−1.09	$<1\times 10^{-16}$
α	1.0021	0.0008	1.0005	1.0036	0.0084
Hypothetical Estimand 1	−15.17	0.42	−16.00	−14.34	$<1\times 10^{-16}$
Adherence adjustment in both arms: θ=(β,α,γ) (C=$Y_{0}$)					
β	−1.63	0.07	−1.76	−1.49	$<1\times 10^{-16}$
α	0.9958	0.0009	0.9940	0.9976	6$\times 10^{-6}$
γ	−2.00	0.19	−2.36	−1.63	$<1\times 10^{-16}$
Treatment arm contrast (a)	−16.62	0.45	−17.51	−15.74	$<1\times 10^{-16}$
Placebo arm contrast (b)	−1.99	0.19	−2.36	−1.63	$<1\times 10^{-16}$
Hypothetical Estimand 2: (a)–(b)	−14.63	0.41	−15.44	−13.82	$<1\times 10^{-16}$
Adherence adjustment in treatment arm only: θ=(β,α) (C=Full set)					
β	−1.17	0.04	−1.25	−1.09	$<1\times 10^{-16}$
α	1.0022	0.0008	1.0007	1.0037	0.0049
Hypothetical Estimand 1:	−15.25	0.42	−16.09	−14.42	$<1\times 10^{-16}$
Adherence adjustment in both arms: θ=(β,α,γ) (C=Full set)					
β	−1.60	0.056	−1.71	−1.49	$<1\times 10^{-16}$
α	0.9962	0.0008	0.9947	0.9978	3$\times 10^{-6}$
γ	−1.89	0.10	−2.09	−1.68	$<1\times 10^{-16}$
Treatment arm contrast (a)	−16.63	0.44	−17.49	−15.77	$<1\times 10^{-16}$
Placebo arm contrast (b)	−1.88	0.10	−2.08	−1.68	$<1\times 10^{-16}$
Hypothetical Estimand 2: (a)–(b)	−14.75	0.41	−15.55	−13.94	$<1\times 10^{-16}$

### 3.1 Adherence Adjustment in Treatment Arm Only

**FIGURE 3:**
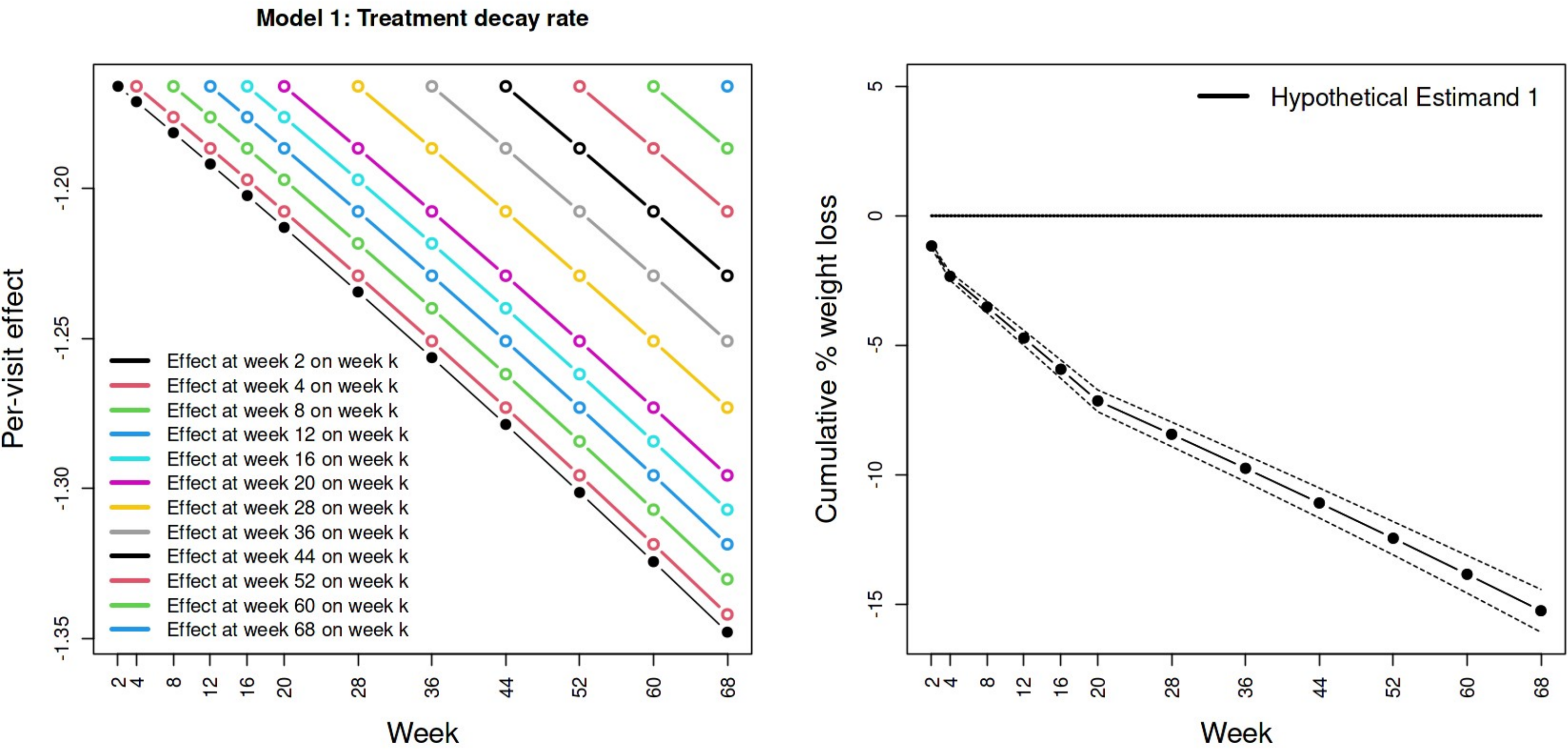
Left: Estimated average causal effects of treatment administered at each time point on weight at subsequent future time points obtained from Model 1. Right: Hypothetical Estimand 1 estimates and 95% confidence intervals for the effect of receiving treatment in full versus none up to week k, for k∈{2,…,68}. The week 68 point estimate and 95% confidence interval corresponds to Hypothetical Estimand 1.

When modeling nonadherence in the treatment arm only, and incorporating the full covariate vector C into the analysis, structural model (6) estimates an immediate effect of treatment at time point k on weight at week k as a reduction of 1.17%. Surprisingly, we see that the decay parameter α governing the effect of earlier treatment on later weight is slightly larger than 1. Figure 3 (left) shows that treatment taken at week 2 is inferred to induce a 1.35% weight reduction at week 68 (bottom‐right black dot). This interpretation is arguably biologically implausible. Nevertheless, Figure 3 (right) shows the point estimate and 95% confidence intervals for the weight loss trajectory under full treatment adherence implied by model (6) at each of the 12 time points in the trial. Hypothetical Estimand 1, the mean difference in percentage weight loss under full adherence to treatment at week 68, is estimated to be a 15.25% reduction (95% CI: −14.43%,−16.07%). In this case, full covariate adjustment is seen to produce near identical point estimates and confidence intervals compared with using baseline weight alone (Table 1).

### 3.2 Adherence Adjustment in Treatment and Placebo Arms

**FIGURE 4:**
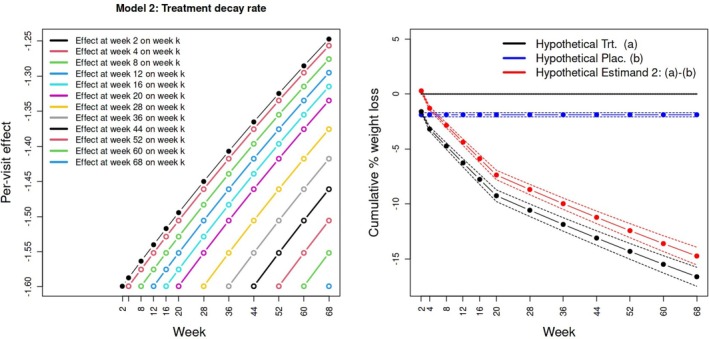
Left: Estimated average causal effects of treatment administered at each time point on weight at subsequent future time points under Model 2. Right: Estimand Hypothetical Estimands and 95% confidence intervals for the effect of receiving (a) treatment in full versus none and (b) placebo in full versus none up to week k, for k∈{2,…,68}. The difference between the treatment and placebo trajectories is shown in red, with the week 68 point estimate and 95% confidence interval of the red curve corresponds to Hypothetical Estimand 2.

Table 1 and Figure 4 show the results of fitting structural causal model (13) accounting for nonadherence in both the treatment and placebo arms. Under this model, and using full covariate adjustment, the causal effect of taking treatment at week k is a (larger) 1.6% reduction in weight at week k. To counter‐balance this, the decay parameter α is estimated to be less than 1, meaning that effect of taking treatment at week k on weight in future weeks follows a more plausible diminishing trend (Figure 4 left). For example, the total weight loss at week 68 that is attributable to treatment at week 2 is ≈ −1.25%. In the placebo arm, the constant instantaneous placebo effect parameter γ is estimated to be −1.89%.

Figure 4 (right) shows three implied weight loss trajectories: first, the weight change in the treatment arm under full adherence versus no adherence (black line, contrast (a) in Table 1; second, the weight change in the placebo arm under full adherence versus no adherence (blue line, contrast (b) in Table 1); and thirdly, the difference between (a) and (b) (red line), whose 12th and final time‐point at week 68 is an estimate for Hypothetical Estimand 2. At week 68, our model implies that there would have been a 16.63% weight reduction in the treatment arm, leading to an estimate for Hypothetical Estimand 2 of −14.75%, 95% C.I. (−13.94%,−15.55%). In this case, full covariate adjustment is seen to produce similar point estimates and confidence intervals for Hypothetical Estimand 2 compared with using baseline weight alone (Table 1). However, standard errors for $\hat{\beta },\hat{\alpha }$ and $\hat{\gamma }$ are noticeably smaller when all covariates are used.

## 4 Discussion

In this article, we propose a structural mean modeling framework for targeting hypothetical estimands in the landmark STEP 1 trial. It uses randomization as an IV and is able to fully exploit longitudinal data on the weight loss outcome. As such, it represents a significant extension to previously proposed IV methods within the estimand framework [20]. Our decay model formulation developed specifically for STEP 1 is nevertheless well suited to modeling general outcome trajectories, whose complex dynamics are affected by an individual's complete adherence history. Extensions of the methodology beyond a continuous outcome to the binary or time‐to‐event setting will be pursued as further work. We view it as an attractive alternative to the approach of Olarte Parra et al. [7], which can incorporate time‐varying covariates (including previous adherence and outcome measures) into estimators based on inverse probability weighting or G‐computation, due to not relying on the sequential ignorability assumption nor being sensitive to positivity violations. In a broader sense, lack of sensitivity to positivity violation is important because clinical trials can face strongly selective intercurrent events (e.g., treatment discontinuation, rescue treatments) which may be (near‐)deterministically indicated when severe adverse events, such as disease progression, occur.

The suggested robustness of our IV approach comes at the expense of modeling assumptions on the functional form of the causal effect and who it applies to: specifically Equations (5) and (6) for Hypothetical Estimand 1, and Equations (5), (12), and (13) for Hypothetical Estimand 2. These assumptions are partially untestable, but we nonetheless view them to be weaker than assuming that measurements are available on all prognostic factors of the outcome that influence the decision to discontinue treatment, and that the association of these measurements with treatment discontinuation or the outcome has been correctly modeled. Furthermore, although our modeling assumptions are guaranteed to hold under the null hypothesis of no treatment effect, meaning that the proposed approach is guaranteed to deliver robust tests of that null hypothesis, this is not the case for approaches that rely on sequential ignorability.

Our focus in this study was on Hypothetical Estimands, which attempt to quantify effects of interventions on the full trial population. An alternative but closely related strategy mentioned in ICH E9 guidance [6] is to target a Principal Stratum estimand. This general strategy focuses on the effect of (randomized) treatment assignment in a specific participant subgroup defined by their potential adherence to one or more treatments in the trial [20]. For example, Qu et al. (2020) [25] propose two Principal Strata of interest when conducting a general analysis within the Estimand framework: first, participants who would fully adhere to randomized treatment in both arms; and second, participants who would fully adhere to the active treatment if randomized to receive it, but who may or may not adhere to placebo if randomized to receive it. Strong assumptions are needed to learn the effect for these patient strata, because the observed data do not directly enable one to learn *which* participants would be adherent to active treatment as well as placebo. For example, when defining Hypothetical Estimand 2, we do not claim that the group of full treatment adherers is the same as the group of full placebo adherers, nor that we could identify an effect for any single individual who would be a full treatment adherer *and* a full placebo adherer. Qu et al. [25] and others [26, 37] identify treatment effects in these strata by assuming, amongst other things, the availability of post‐treatment measurements Z (e.g., safety outcomes) with counterfactual values Z(R=0) and Z(R=1) that are conditionally independent given baseline covariates. Future work will focus on hypothetical estimands for observable participant subgroups defined at an intermediate time (e.g., week 20 treatment “responders” in the context of STEP 1). We believe these can be identified with minor adaptations of the causal model considered here, thereby avoiding such strong assumptions.

We view our present proposal as useful starting point to influence the development of more sophisticated approaches. For example, the incorporation of covariates as causal effect modifiers to improve the plausibility of assumptions (5) and (12). In addition, to further improve its biological plausibility when applied to weight loss data beyond 68 weeks, we will look to incorporate a weight loss “plateau feature” into the model, as observed in the STEP 5 trial that compared two years of semaglutide treatment to placebo [38]. Extensions of our approach to the optimal dynamic treatment regime setting, as in [39], would also be highly desirable.

In estimating Equations (7) and (14), we used the identity matrix for ∑, but a more complex choice that can in theory deliver better efficiency is the residual covariance matrix of the predicted adherence‐free potential outcomes. This quantity depends on the unknown model parameters and must therefore be updated at each iteration of the score function minimization. An advantage of this is that the score function then follows a known $\chi^{2}$ distribution. As further work, we plan to incorporate this into our software, as well as developing a framework to assess the goodness of fit of a range possible causal models, with a view to further improving the performance and power of our method.

## Conflicts of Interest

Stijn Vansteelandt serves as a consultant for Novo Nordisk. The other authors declare no conflicts of interest.

## Supporting information


**Data S1.** Supporting Information.

## Figures and Tables

**FIGURE A1:**
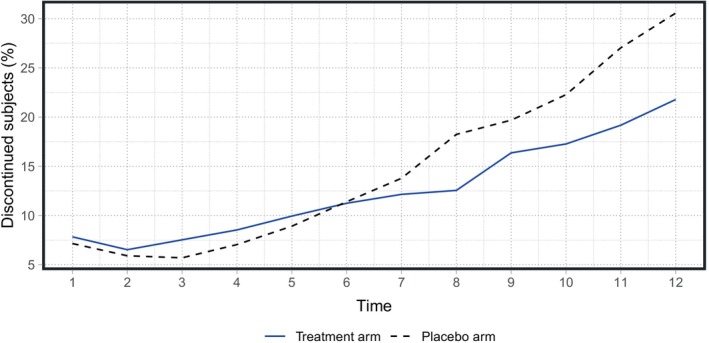
Adherence rates in the treatment and placebo arm over the 12 time periods of the trial for a representative single data set.

## Data Availability

Data sharing is not applicable to this article, as no new data were created or analyzed in this study.

## Appendix A Monte Carlo Simulations

We perform Monte Carlo simulations to sanity check the validity of our proposed G‐estimation method and sandwich variance formula (15) for quantifying Hypothetical Estimand 1 and 2. To mimic the setup of STEP 1, we generate simulated data sets with n=1961 individuals across K=12 time points. For simplicity, we make the time intervals equally spaced, so that $t_{k}=k$, k=1,…,12. For each individual i, their initial treatment assignment $R_{i}$ is generated from a Bernoulli distribution with $Pr(R_{i}=1)=\frac{1}{2}$, with $R_{i}=1$ being the experimental treatment and $R_{i}=0$ being placebo. For individual i at time point k, we denote their adherence status by $A_{i,k}$, whereby $A_{i,k}=1$ indicates that they take the real (or sham in case of placebo) injection as intended, and $A_{i,k}=0$ if they do not. We can then write the actual adherence status for individual i at time point k as $A_{i,k}R_{i}$ in the treatment arm or $A_{i,k}\left(1-R_{i}\right)$ in the placebo arm.

### Adherence Status Generation

Let $PT_{i,k}$ ($PP_{i,k}$) be the probability of $A_{i,k}=1$ for individuals in the treatment (placebo) arm. $PT_{i,k}$ is determined by the following logit model:

$$PT_{i,k}=\frac{e^{\left(\eta_{0}+\eta_{1}A_{i,k-1}+\eta_{2}Y_{i,k-1}+\eta_{k}k+U_{i,k}\right)}}{1+e^{\left(\eta_{0}+\eta_{1}A_{i,k-1}+\eta_{2}Y_{i,k-1}+\eta_{k}k+U_{i,k}\right)}}$$

where

- $A_{i,k-1}$ and $Y_{i,k-1}$ are the adherence status and outcome at the previous time point;
- $U_{i,k}$ is an unobserved serially correlated confounder generated by the following auto‐regressive AR(1) process:
  $$U_{i,k}=0.98U_{i,k-1}+N(0.0.2^{2})$$
  where $U_{i,k-1}=0$ with k=1;
- $\eta_{0}\ldots \eta_{k}$ are fixed parameters.

When k=1, we set $A_{i,k-1}=Y_{i,k-1}=0$ and ignore the time effect by setting $\eta_{k}=0$. We set $\eta_{0}=3$, $\eta_{1}=0.2$, $\eta_{2}=-0.1$ and $\eta_{k}=-0.2$. These coefficients imply that an individual is more likely to adhere if they have previously and if they experience a larger weight loss. It also induces a decrease in the probability of adherence over time. Similarly, we generate adherence status variables $A_{i,k}$ in the placebo arm from a Bernoulli distribution with

$$PP_{i,k}=\frac{e^{\left(\pi_{0}+\pi_{1}A_{i,k-1}+\pi_{2}Y_{i,k-1}+\pi_{k}k+U_{i,k}\right)}}{1+e^{\left(\pi_{0}+\pi_{1}A_{i,k-1}+\pi_{2}Y_{i,k-1}+\pi_{k}k+U_{i,k}\right)}}$$

where $\pi_{0}=3$, $\pi_{1}=0.3$, $\pi_{2}=-0.25$ and $\pi_{k}=-0.2$.

### Outcome Generation

For transparent estimation of the parameters furnishing Hypothetical Estimand 1, we first generate outcome $Y_{i,k}$ from the following model, assuming the placebo effect is zero:

(A1)
$$Y_{i,k}=\overset{k}{\underset{t=1}{\sum }}\beta \alpha^{k-t}A_{i,t}R_{i}+U_{i,k}$$

For transparent estimation of the parameters furnishing Hypothetical Estimand 2, we generate $Y_{i,k}$ from the following model which incorporates a non‐zero placebo effect:

(A2)
$$Y_{i,k}=\overset{k}{\underset{t=1}{\sum }}\beta \alpha^{k-t}A_{i,t}R_{i}+\gamma A_{i,k}(1-R_{i})+U_{i,k}$$

where γ is set to −0.9. For simplicity, we do not include baseline covariates in the adherence or outcome models. They can, however, be viewed as residuals after partialing out baseline covariates (see Section 2.6.2).

The proportion of non‐adherers within each trial arm and at each of the 12 time points, is shown for a single representative simulated data set in Figure A1 below.

Over 1000 simulated data sets, we estimated causal parameters β and α using the G‐estimation method elaborated in Section 2.3 with outcome data generated by Equation (A1). Using 1000 simulated data sets from outcome model (A2), we then estimated β, α and γ using the G‐estimation strategy in Section 2.5 for targeting Hypothetical Estimand 2. In both cases, we set ∑ as the 12×12 identity matrix. The results are presented below in Table A1. We report three summary statistics across the 1000 simulations: “*Estimate*”: the mean values of the parameter estimates; “*Empirical SE*”: the standard deviation of the parameter estimates; “*Sandwich SE*” is the mean standard error of the estimates calculated from the sandwich variance formula in Section 2.6.1. For both models, the G‐estimation method works as intended, with mean point estimates close to the ground truth, and empirical standard errors matching those obtained from the sandwich variance formula.

**TABLE A1:** Simulation Results: Mean Point Estimates, Empirical Standard Errors and Mean Sandwich Variance Standard Errors of the Causal Parameters Furnishing Hypothetical Estimand 1 and 2, Over 1000 Replications.

	β	α	γ
**Truth**	−1.1	0.95	−0.9
**Estimand 1:**			
Estimate	−1.102	0.949	—
Empirical SE	0.017	0.003	—
Sandwich SE	0.017	0.002	—
**Estimand 2:**			
Estimate	−1.103	0.950	−0.907
Empirical SE	0.009	0.001	0.051
Sandwich SE	0.009	0.001	0.052

R code to reproduce the simulation study is available in *Online Supporting Information*.

## References

[1] C. C. Curioni and P. M. Lourenço , “Long‐Term Weight Loss After Diet and Exercise: A Systematic Review,” International Journal of Obesity 29 (2005): 1168–1174. 15925949 10.1038/sj.ijo.0803015

[2] T. M. Frayling , N. J. Timpson , M. N. Weedon , et al., “A Common Variant in the FTO Gene Is Associated With Body Mass Index and Predisposes to Childhood and Adult Obesity,” Science 316 (2007): 889–894. 17434869 10.1126/science.1141634 PMC2646098

[3] A. Patel , D. Gill , D. Shungin , et al., “Robust Use of Phenotypic Heterogeneity at Drug Target Genes for Mechanistic Insights: Application of Cis‐Multivariable Mendelian Randomization to GLP1R Gene Region,” Genetic Epidemiology 48, no. 4 (2024): 151–163, 10.1002/gepi.22551. 38379245 PMC7616158

[4] J. Nudel and V. M. Sanchez , “Surgical Management of Obesity,” Metabolism 92 (2019): 206–216. 30576688 10.1016/j.metabol.2018.12.002

[5] J. P. H. Wilding , R. L. Batterham , S. Calanna , et al., “Once‐Weekly Semaglutide in Adults With Overweight or Obesity,” New England Journal of Medicine 18 (2021): 989–1002. 10.1056/NEJMoa2032183 33567185

[6] “ICH E9(R1) Addendum on Estimands and Sensitivity Analysis in Clinical Trials to the Guideline on Statistical Principles for Clinical Trials,” 2017.European Medicines Agency.

[7] C. Olarte Parra , R. M. Daniel , and J. W. Bartlett , “Hypothetical Estimands in Clinical Trials: A Unification of Causal Inference and Missing Data Methods,” Statistics in Biopharmaceutical Research 15 (2022): 421–432. 37260584 10.1080/19466315.2022.2081599 PMC10228513

[8] J. M. Robins and A. A. Tsiatis , “Correcting for Non‐compliance in Randomized Trials Using Rank Preserving Structural Failure Time Models,” Communications in Statistics ‐ Theory and Methods 20 (1991): 2609–2631.

[9] J. Bowden , S. Seaman , X. Huang , and I. R. White , “Gaining Power and Precision by Using Model‐Based Weights in the Analysis of Late Stage Cancer Trials With Substantial Treatment Switching,” Statistics in Medicine 30 (2016): 1423–1440. 10.1002/sim.6801 PMC4871231 26576494

[10] E. Goetghebeur and K. Lapp , “The Effect of Treatment Compliance in a Placebo‐Controlled Trial: Regression With Unpaired Data,” Journal of the Royal Statistical Society, Series C 46 (1997): 351–364.

[11] S. Greenland , S. Lanes , and M. Jara , “Estimating Effects From Randomized Trials With Discontinuations: The Need for Intent‐To‐Treat Design and G‐Estimation,” Clinical Trials 5 (2008): 5–13. 18283074 10.1177/1740774507087703

[12] K. Fischer , E. Goetghebeur , B. Vrijens , and I. White , “A Structural Mean Model to Allow for Noncompliance in a Randomized Trial Comparing 2 Active Treatments,” Biostatistics 12 (2011): 247–257. 20805286 10.1093/biostatistics/kxq053 PMC3062146

[13] J. Cuzick , R. Edwards , and N. Segnan , “Adjusting for Non‐compliance and Contamination in Clinical Trials,” Statistics in Medicine 2 (1997): 808–840. 10.1002/(sici)1097-0258(19970515)16:9<1017::aid-sim508>3.0.co;2-v 9160496

[14] C. Frangakis and D. Rubin , “Principal stratification in causal inference,” Biometrics 58 (2002): 21–29. 11890317 10.1111/j.0006-341x.2002.00021.x PMC4137767

[15] J. Lin , T. Ten Have , and M. Elliot , “Longitudinal Nested Compliance Class Model in the Presence of Time‐Varying Noncompliance,” Journal of the American Statistical Association 102 (2008): 462–473.

[16] G. D. Smith and S. Ebrahim , “‘Mendelian Randomization’: Can Genetic Epidemiology Contribute to Understanding Environmental Determinants of Disease?,” International Journal of Epidemiology 32 (2003): 1–22. 12689998 10.1093/ije/dyg070

[17] J. Bowden and S. Vansteelandt , “Mendelian Randomization Analysis of Case‐Control Data Using Structural Mean Models,” Statistics in Medicine 30 (2011): 678–694. 21337362 10.1002/sim.4138

[18] A. Brookhart and S. Schneeweiss , “Preference‐Based Instrumental Variable Methods for the Estimation of Treatment Effects: Assessing Validity and Interpreting Results,” International Journal of Biostatistics 3, no. 1 (2007): 1–23, 10.2202/1557-4679.1072. 19655038 PMC2719903

[19] L. M. Güdemann , K. G. Young , N. J. M. Thomas , et al., “Safety and Effectiveness of SGLT2 Inhibitors in a UK Population With Type 2 Diabetes and Aged Over 70 Years: An Instrumental Variable Approach,” Diabetologia 67 (2024): 1817–1827. 38836934 10.1007/s00125-024-06190-9 PMC11410842

[20] J. Bowden , B. Bornkamp , E. Glimm , and F. Bretz , “Connecting Instrumental Variable Methods for Causal Inference to the Estimand Framework,” Statistics in Medicine 40 (2021): 5605–5627. 34288021 10.1002/sim.9143

[21] A. Ying and E. J. T. Tchetgen , “Structural Cumulative Survival Models for Estimation of Treatment Effects Accounting for Treatment Switching in Randomized Experiments,” Biometrics 79 (2023): 1597–1609. 35665918 10.1111/biom.13704

[22] H. Michiels , A. Vandebosch , and S. Vansteelandt , “Adjusting for Time‐Varying Treatment Switches in Randomized Clinical Trials: The Danger of Extrapolation and How to Address It,” Statistics in Biopharmaceutical Research 17 (2024): 54–66.

[23] N. Yende‐Zuma , H. Mwambi , and S. Vansteelandt , “Adjusting the Effect of Integrating Antiretroviral Therapy and Tuberculosis Treatment on Mortality for Noncompliance: A Time‐Varying Instrumental Variables Analysis,” Epidemiology 30 (2019): 197–203. 30720587 10.1097/EDE.0000000000000923 PMC6367706

[24] H. Michael , Y. Cui , S. Lorch , and E. Tchetgen Tchetgen , “Instrumental Variable Estimation of Marginal Structural Mean Models for Time‐Varying Treatment,” Journal of the American Statistical Association 119 (2024): 1240–1251. 10.1080/01621459.2023.2183131 PMC13309177 42368411

[25] Y. Qu , H. Fu , J. Luo , and S. J. Ruberg , “A General Framework for Treatment Effect Estimators Considering Patient Adherence,” Statistics in Biopharmaceutical Research 12 (2020): 1–18.

[26] J. Luo , S. J. Ruberg , and Y. Qu , “Estimating the Treatment Effect for Adherers Using Multiple Imputation,” Pharmaceutical Statistics 21 (2022): 525–534. 34927339 10.1002/pst.2184

[27] I. R. White , S. Walker , and A. Babiker , “Strbee: Randomization‐Based Efficacy Estimator,” Stata Journal 2 (2002): 140–150.

[28] J. M. Robins , “Correcting for Non‐compliance in Randomized Trials Using Structural Nested Mean Models,” Communications in Statistics ‐ Theory and Methods 23 (1994): 2379–2412.

[29] S. Vansteelandt and M. Joffe , “Structural Nested Models and G‐Estimation: The Partially Realized Promise,” Statistical Science 29 (2014): 707–731.

[30] J. Pearl , Causality (Cambridge university press, 2009).

[31] R. Bowden and D. Turkington , Instrumental Variables (Cambridge University Press, 1990).

[32] M. Hernán and J. Robins , Causal Inference: What if (Chapman & Hall/CRC Press, 2020).

[33] S. Vansteelandt and E. Goetghebeur , “Causal Inference With Generalized Structural Mean Models,” Journal of the Royal Statistical Society, Series B: Statistical Methodology 65 (2003): 817–835.

[34] W. Newey and D. McFadden , “Chapter 36: Large Sample Estimation and Hypothesis Testing,” in Handbook of Econometrics (Elsevier Science, 1994).

[35] I. R. White , P. Royston , and A. M. Wood , “Multiple Imputation Using Chained Equations: Issues and Guidance for Practice,” Statistics in Medicine 30 (2011): 377–399. 21225900 10.1002/sim.4067

[36] D. B. Rubin , Flexible Imputation of Missing Data, 2nd ed. (Chapman and Hall/CRC, 2018).

[37] B. Bornkamp and G. Bermann , “Estimating the Treatment Effect in a Subgroup Defined by an Early Post‐Baseline Biomarker Measurement in Randomized Clinical Trials With Time‐To‐Event Endpoint,” Statistics in Biopharmaceutical Research 12 (2020): 19–28.

[38] W. T. Garvey , R. L. Batterham , M. Bhatta , et al., “Two‐Year Effects of Semaglutide in Adults With Overweight or Obesity: The STEP 5 Trial,” Nature Medicine 28 (2022): 2083–2091. 10.1038/s41591-022-02026-4 PMC9556320 36216945

[39] I. Bhattacharya , A. Ertefaie , K. G. Lynch , J. R. McKay , and B. A. Johnson , “Nonparametric Bayesian Q‐Learning for Optimization of Dynamic Treatment Regimes in the Presence of Partial Compliance,” Statistical Methods in Medical Research 32 (2023): 1649–1663. 37322885 10.1177/09622802231181223 PMC11625440

